# Translational arrest and mRNA decay are independent activities of alphaherpesvirus virion host shutoff proteins

**DOI:** 10.1099/jgv.0.001976

**Published:** 2024-04-04

**Authors:** Lucy Eke, Alistair Tweedie, Sophie Cutts, Emma L. Wise, Gillian Elliott

**Affiliations:** 1 Section of Virology, Department of Microbial Sciences, School of Biosciences, University of Surrey, Guildford, UK

**Keywords:** alphaherpesviruses, BHV1, EHV1, endoribonuclease, G3BP1, HSV1, MDV, PABPC1, poly(A)+ FISH, stress granules, vhs, VZV

## Abstract

The herpes simplex virus 1 (HSV1) virion host shutoff (vhs) protein is an endoribonuclease that regulates the translational environment of the infected cell, by inducing the degradation of host mRNA via cellular exonuclease activity. To further understand the relationship between translational shutoff and mRNA decay, we have used ectopic expression to compare HSV1 vhs (vhsH) to its homologues from four other alphaherpesviruses – varicella zoster virus (vhsV), bovine herpesvirus 1 (vhsB), equine herpesvirus 1 (vhsE) and Marek’s disease virus (vhsM). Only vhsH, vhsB and vhsE induced degradation of a reporter luciferase mRNA, with poly(A)+ *in* *situ* hybridization indicating a global depletion of cytoplasmic poly(A)+ RNA and a concomitant increase in nuclear poly(A)+ RNA and the polyA tail binding protein PABPC1 in cells expressing these variants. By contrast, vhsV and vhsM failed to induce reporter mRNA decay and poly(A)+ depletion, but rather, induced cytoplasmic G3BP1 and poly(A)+ mRNA- containing granules and phosphorylation of the stress response proteins eIF2α and protein kinase R. Intriguingly, regardless of their apparent endoribonuclease activity, all vhs homologues induced an equivalent general blockade to translation as measured by single-cell puromycin incorporation. Taken together, these data suggest that the activities of translational arrest and mRNA decay induced by vhs are separable and we propose that they represent sequential steps of the vhs host interaction pathway.

## Introduction

A wide range of viruses, including influenza viruses, coronaviruses and herpesviruses express endoribonucleases to help regulate the mRNA environment in the infected cell and favour virus over host translation [1,6]. In the case of the alphaherpesvirus herpes simplex virus 1 (HSV1), the product of the UL41 gene, named the virion host shutoff protein (vhs), is well documented as an endoribonuclease that functions not only during infection [7,9], but also when expressed in isolation, where it has been shown to abrogate the expression of reporter proteins such as beta-galactosidase and luciferases [10,14]. vhs is proposed to induce the degradation of mRNA [15,17] by binding to the cellular translation initiation machinery through the eIF4A and eIF4H components of the eIF4F cap-binding complex, resulting in mRNA cleavage [18,21]. It is hypothesized that as for the KSHV SOX protein, the cellular exonuclease XRN1 is subsequently recruited to cleave mRNA and accelerate its decay in the cytoplasm [22]. An additional consequence of vhs-induced mRNA decay is the accumulation of polyA binding protein (PABPC1) in the nucleus [13,23, 24]. Ordinarily, PABPC1 cycles between the nucleus and cytoplasm on the polyA tails of exporting mRNAs exhibiting a steady-state cytoplasmic localization [25,26]. When this balance is disturbed through enhanced mRNA decay in the cytoplasm, PABPC1 accumulates in the nucleus along with other RNA binding proteins [27] resulting in the hyperadenylation of mRNA and a subsequent block to mRNA export [23,26]. Recently, vhs has also been shown to induce the degradation of double-stranded RNA contributing to the inhibition of the cellular protein kinase R (PKR) response seen in virus infection [28], and providing an explanation for the documented induction of stress granules in cells infected with vhs knockout viruses [29,30]. It is suggested that in the absence of vhs, dsRNA forms as a consequence of transcription of both strands of the HSV1 genome, inducing the phosphorylation of PKR and eIF2α, the stalling of translation and induction of stress granules [31], thereby shutting down viral as well as cellular protein synthesis.

**Fig. 1.**
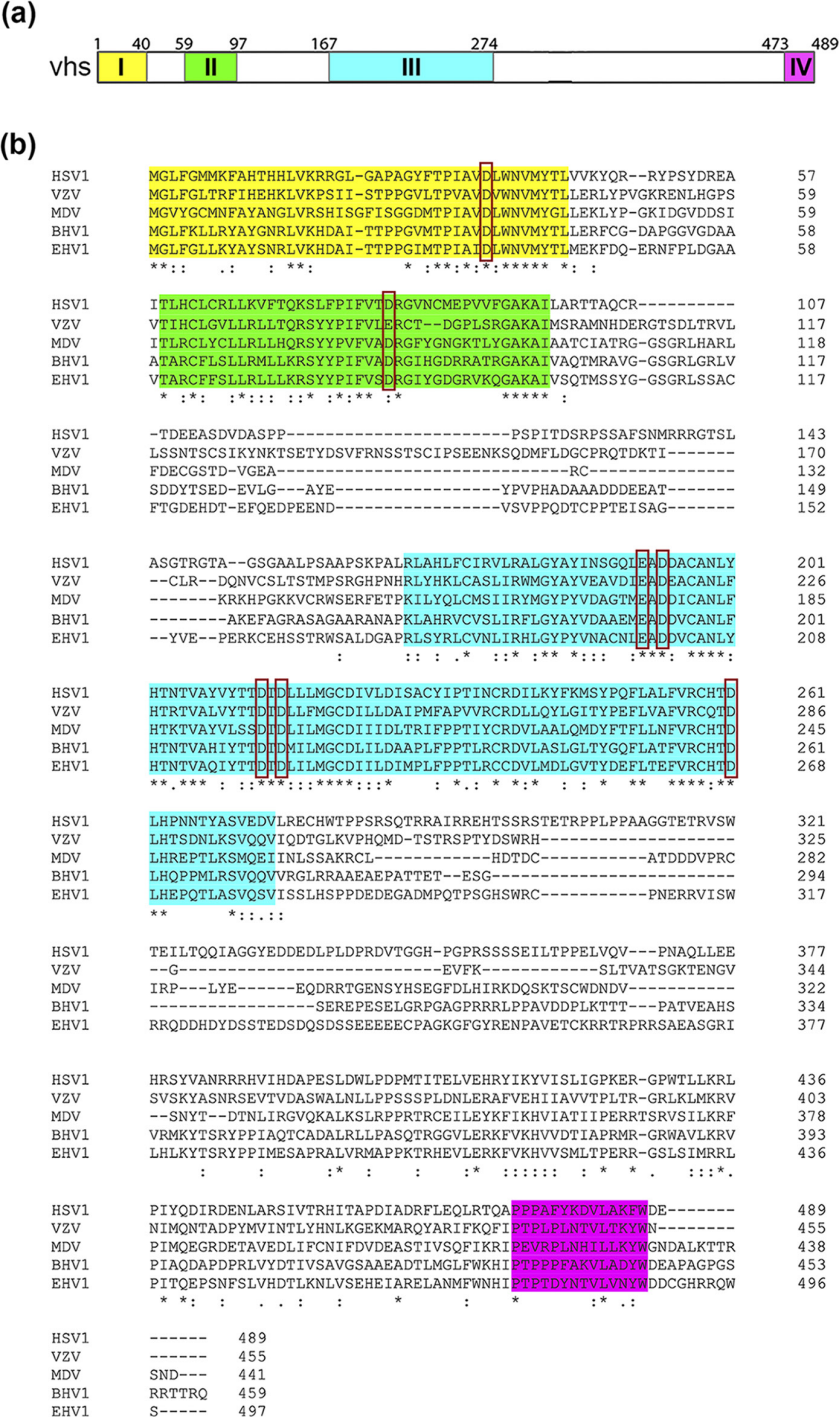
Amino acid alignment of vhs homologues from HSV1, VZV, BHV1, EHV1 and MDV. (**a**) Line drawing of the HSV1 vhs open reading frame. Four conserved areas are coloured and based on [32]. (**b**) A multiple sequence alignment for vhs homologues from HSV1 (YP_009137116), VZV (NP_040140), MDV (YP_001033970), BHV1 (NP_045317) and EHV1 (YP_053064) was performed using Clustal Omega with default settings. Red boxes indicate conserved acidic residues proposed to be important for metal ion binding [18].

The UL41 gene is present in all alphaherpesviruses, and alignment of the proteins has previously identified four conserved domains across the protein [32]. In HSV1 vhs, these regions (Fig. 1a) have been shown to be important for vhs activity [12,33]. The activity of the other alphaherpesvirus vhs proteins is poorly defined in comparison to HSV1 vhs: homologues from pseudorabies virus and bovine herpesvirus 1 (BHV1) have been shown to function when expressed either ectopically or in the virus [34,38]; equine herpesvirus 1 (EHV1) and Marek’s disease virus (MDV) homologues appear to be active when expressed in isolation but not in the context of virus infection [39,41]; and there are conflicting reports on the functionality of varicella zoster virus (VZV) vhs [42,43]. Although the metal ion-binding residues in the active site are conserved across the vhs homologues (Fig. 1b, red boxes [18]), the overall level of identity between these proteins ranges from only 33–51 % (Table 1), and hence there is scope for differential activity amongst the homologues.

**Table 1.** Identity matrix between the homologues of vhs used in this study

	HSV1	VZV	MDV	BHV1	EHV1
**HSV1**	**100**	33.57	33.01	35.75	33.48
**VZV**	33.57	**100**	38.44	40.77	40.46
**MDV**	33.01	38.44	**100**	41.35	39.82
**BHV1**	35.75	40.77	41.35	**100**	51.1
**EHV1**	33.48	40.46	39.82	51.1	**100**

Here we have evaluated the relative activity of VZV (vhsV), BHV1 (vhsB), EHV1 (vhsE) and MDV (vhsM) homologues in relation to that from HSV1 (vhsH) and have compared their behaviour when expressed in isolation of virus infection. We show that vhsB and vhsE behave in a similar fashion to vhsH, abrogating gene expression from a reporter plasmid and inducing the degradation of its mRNA. They also induced a general depletion of cytoplasmic polyadenylated (poly(A)+) RNA and the relocalization of PABPC1 to the nucleus; of note, vhsB was seen to be the most highly active vhs homologue. By contrast, vhsV did not degrade the reporter mRNA or deplete the cytoplasm of poly(A)+ RNA, but instead caused the formation of G3BP1-containing RNA granules in expressing cells; vhsM did not degrade mRNA but exhibited PABPC1 relocalization characteristics of both vhsH and vhsV. Moreover, despite the lack of mRNA decay in cells expressing vhsV or vhsM, single-cell puromycin incorporation revealed that all five vhs homologues were able to induce a general blockade to cellular translation, suggesting that translational arrest is independent of mRNA decay. These data highlight the benefit of studying the activity of a virus protein from different family members to unravel important mechanistic detail.

## Methods

### Cells and plasmids

HeLa cells were cultured in DMEM supplemented with 10 % foetal bovine serum (Invitrogen). Plasmids pEGFPC1 and pEGFPN1, expressing GFP under the control of the HCMV major immediate-early promoter were obtained from Clontech. The construction of plasmids expressing vhs from HSV1 strain17 as vhs-GFP and untagged pcvhs, or non-functional untagged vhs (vhs*) containing a single point mutation (R257C) have been described previously [13,44]. vhsB-GFP, vhsE-GFP and vhsM-GFP were constructed by PCR amplification of the following; UL41 from BHV1 strain P8-2; ORF 19 from EHV1 strain Ab4; and 054 from MDV strain RB1B followed by insertion into pEGFPN1. The VZV ORF17 gene was synthesized from reference sequence MH709372.1 and inserted into pEGFPN1. Untagged versions were constructed by replacing the GFP open reading frame with a stop codon.

### Antibodies

Commercial antibodies used in this study were α-tubulin (Sigma), GFP (Clontech), PABPC1 (Santa Cruz) G3BP1 (Sigma) and Puromycin (EMD Millipore).

### Quantitative RT-PCR (RT-qPCR)

Total RNA was extracted from cells using Qiagen RNeasy kit. Excess DNA was removed by incubation with DNase I (Invitrogen) for 15 min at room temperature, followed by inactivation for 10 min at 65 °C in 25 nM of EDTA. Superscript III (Invitrogen) was used to synthesize cDNA using random primers according to manufacturer’s instructions. All RT-qPCR assays were carried out in 96-well plates using MESA Blue qPCR MasterMix Plus for SYBR Assay (Eurogentec). GLuc was amplified using GLuc forward primer CCTACGAAGGCGACAAAGAG and reverse primer TTGTGCAGTCCACACACAGA; GFP was amplified with forward primer GAAGCAGCACGACTTCTTCAA and reverse primer AGTCGATGCCCTTCAGCTC. Cycling was carried out in a Lightcycler (Roche), and GLuc or GFP mRNA levels determined by the ΔΔCt method using 18 s RNA as reference gene (18 s F: CCAGTAAGTGCGGGTCATAAGC; 18 s R: GCCTCACTAAACCATCCAATCGG).

### SDS-PAGE and Western blotting

Protein samples were analysed by SDS-polyacrylamide gel electrophoresis and transferred to nitrocellulose membrane for Western-blot analysis. IRDye secondary antibodies were obtained from LI-COR and Western blots were imaged on a LI-COR Odyssey Imaging system.

### Immunofluorescence

Cells for immunofluorescence were grown on coverslips and fixed with 4 % paraformaldehyde in PBS for 20 min at room temperature, followed by permeabilization with 0.5 % Triton-X100 for 10 min. Fixed cells were blocked by incubation in PBS with 10 % newborn calf serum for 20 min, before the addition of primary antibody in PBS with 10 % serum, and a further 30 min incubation. After extensive washing with PBS, the appropriate Alexafluor conjugated secondary antibody was added in PBS with 10 % serum and incubated for a further 15 min. The coverslips were washed extensively in PBS and mounted in Mowiol containing DAPI to stain nuclei. Images were acquired using a Nikon A1 confocal microscope and processed using ImageJ software.

### Fluorescent *in situ* hybridization (FISH) of RNA

HeLa cells were grown in 2-well slide chambers (Fisher Scientific) and transfected with plasmid using Lipofectamine 2000. Sixteen hours later, cells were fixed for 20 min in 4 % PFA. For poly(A)+ RNA FISH, cold methanol was added to cells for 10 mins followed by 70 % ethanol for 10 mins, then 1M Tris (pH 8) for 5 mins. The cells were then incubated with 1 ng µl^–1^ 5′-labelled Cy3-oligodT in hybridization buffer (0.005 % bovine serum albumin; 1 mg ml^−1^ yeast tRNA; 10 % dextran sulphate; 25 % deionized formamide; in 2 × SSC) at 37 °C for 2 h before sequential washing in 4 × SSC, and 2 × SSC; 0.1 % tritonX100 in 2 × SSC. Cells were mounted in Mowiol containing DAPI to stain nuclei, images were acquired with a Nikon A2 inverted confocal microscope and processed using Adobe Photoshop software.

### *Gaussia* luciferase reporter assay

HeLa cells were transfected with plasmid pCMV-GLuc-1 and increasing amounts of vhs-expressing plasmids using Lipofectamine 2000. After 16 h, the medium was replaced, sampled 4 h later, and chemiluminescence measured by injection of coelenterazine at 1 µg ml^−1^ in PBS and read on a Clariostar plate reader.

### Puromycin incorporation assay

HeLa cells were grown on coverslips before transfection with vhs-expressing plasmids using Lipofectamine 2000. After 16 h, the medium was removed and replaced with medium containing 10 ug ml^−1^ puromycin (Invivogen) and incubated for 5 min at 37 °C. Following incubation, cells were washed once in PBS before fixation for 20 min in 4 % PFA and processing for immunofluorescence.

## Results

### Confirmation of expression of five alphaherpesvirus vhs homologues

**Fig. 2.**
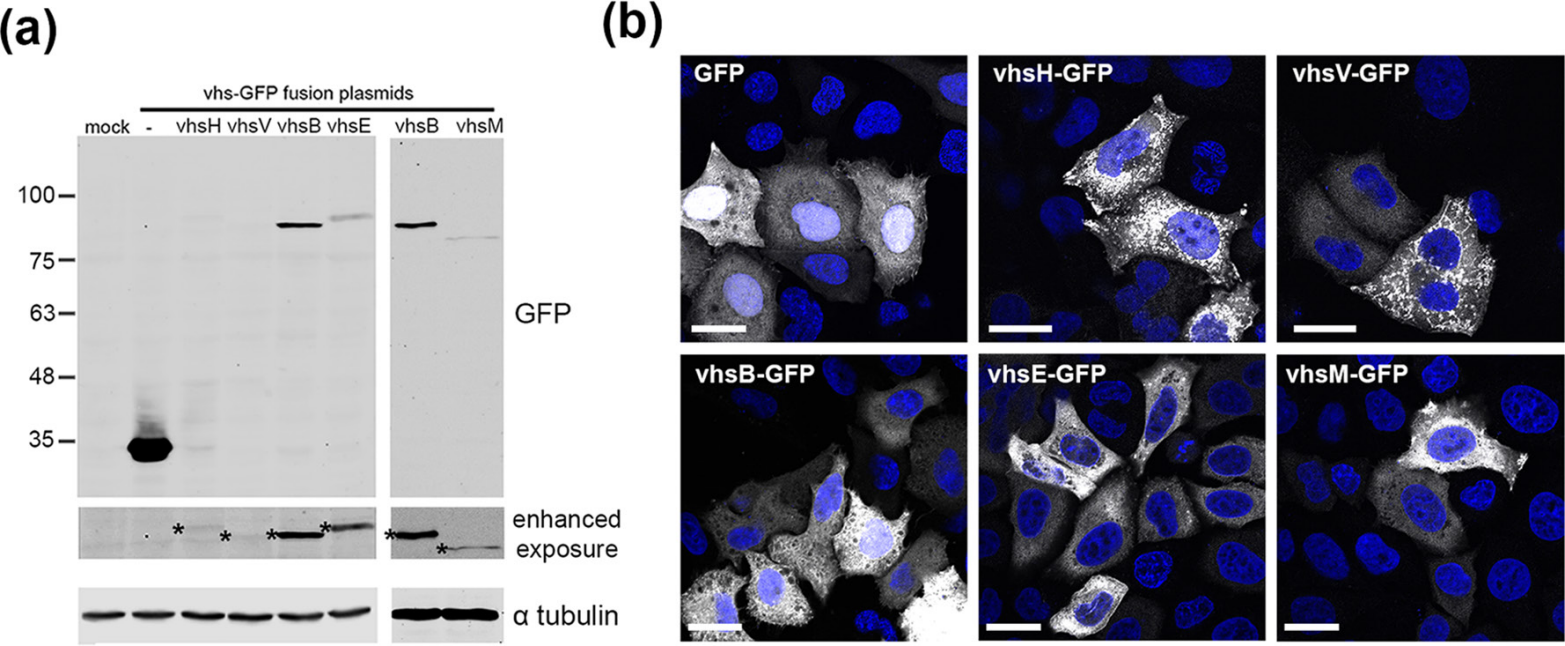
Expression of five vhs homologues as GFP fusion proteins. (**a**) HeLa cells were transfected with plasmids expressing GFP (-) or each of the vhs homologues fused at their C-termini to GFP. After 16 h total cell extracts were prepared and analyzed by SDS-PAGE and Western blotting for GFP or α-tubulin as a loading control. Protein molecular weight markers are shown on left hand side (kDa). * denotes GFP-positive band on blot seen in enhanced exposure. (**b**) HeLa cells grown on coverslips were transfected as for (**a**), fixed after 16 h and nuclei stained with DAPI (blue). GFP fluorescence (white) was imaged using a NikonA1 confocal microscope. Scale bar=20 µm.

To compare the relative expression of five vhs proteins, expression vectors for the vhs open reading frames from HSV1 (vhsH), VZV (vhsV), BHV1 (vhsB), EHV1 (vhsE) and MDV (vhsM), fused at their C-termini to GFP, were constructed as a means of confirming their expression by transient transfection. Of note, we and others have shown that vhsH is expressed at very low levels in transient systems [13,45] while fusion of vhsH to GFP confers this reduced level of expression to the vhsH-GFP fusion protein [44]. We hypothesize that this is due to a combination of a previously identified inhibitory sequence in the vhsH transcript, which blocks translation [13], and the negative feedback of vhsH endoribonuclease activity on its own transcript [13]. Expression and localization of the vhs homologues were compared by transfecting plasmids expressing the GFP-fusion proteins into HeLa cells and analyzing 16 h later by SDS-PAGE and Western blotting for GFP, or by imaging of GFP fluorescence. As shown previously [13,44], a vhsH-GFP fusion protein was expressed at a much lower level than GFP alone, as demonstrated by Western blotting (Fig. 2a). Likewise, the other vhs-GFP fusion proteins were expressed at lower levels than GFP alone (Fig. 2a). Here, vhsV-GFP was barely detectable by Western blotting, but vhsB-GFP, vhsE-GFP and vhsM-GFP were detectable, but still greatly reduced compared to GFP alone (Fig. 2a). Nonetheless, despite this low level of expression, all homologue GFP fusion proteins were detected at the single cell level and were localized predominantly to the cytoplasm (Fig. 2b).

### Differential regulation of a GLuc reporter by the vhs homologues

**Fig. 3.**
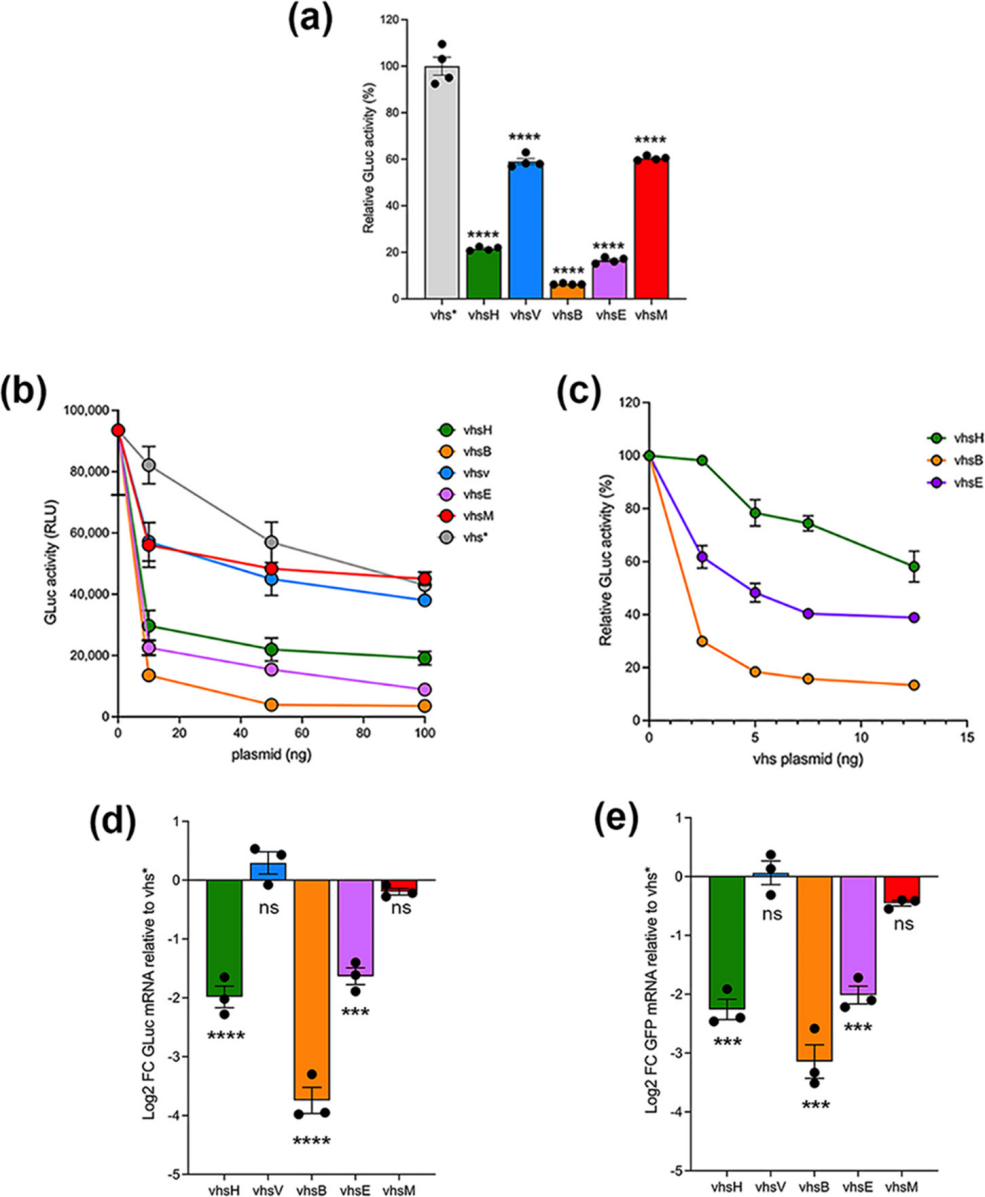
Differential effect of vhs homologues on reporter gene expression. (**a**) HeLa cells were transfected with 50 ng GLuc plasmid alone or in combination with 100 ng untagged vhs homologue encoding plasmids. After 16 h, media was changed and 4 h later media samples were removed and analysed for GLuc activity in a Clariostar plate reader. Values are shown as mean+/-standard error of the mean of four independent experiments. Statistical analysis was carried out using ordinary one-way ANOVA multiple comparisons and comparison made to vhs* values. ****P<0.0001. (**b**) HeLa cells were transfected with GLuc alone or with increasing amounts of plasmids encoding vhs*, vhsH, vhsV, vhsB, vhsE or vhsM. GLuc activity was measured as in (**a**). The mean+/−standard error of the mean from one representative experiment is shown (*n*=3). (**c**) HeLa cells were transfected with GLuc alone or with increasing amounts of plasmids encoding vhsH, vhsB or vhsE. GLuc activity was measured as in (a). The mean+/−standard error of the mean from one representative experiment is shown (*n*=3). (d and e) HeLa cells were transfected as for (**a**) with either (**d**) GLuc- or (**e**) eGFP-expressing plasmid, and after 20 h total RNA was harvested and RTqPCR carried out with primers specific for (d) GLuc or (**e**) eGFP, using 18 s as the reference gene. Differential GLuc or eGFP expression was determined to GLuc or eGFP expression in vhs* expressing cells using the ΔΔCt method. The mean+/−standard error of the mean from one representative experiment is shown (*n*=3). Statistical analysis was carried out using ordinary one-way ANOVA multiple comparisons and comparison made to vhs* values. ns, *P*>0.05. ***P<0.001. ****P<0.0001.

Ectopic expression of vhsH is known to reduce expression of a number of reporter proteins including *Gaussia* luciferase (GLuc), a secreted reporter luminescent protein [13]. As our previous work has revealed that tagging vhsH with even a small epitope can abrogate its activity, the GFP open reading frame was next removed from the vhs-GFP expression vectors above and replaced with a stop codon. These plasmids, now expressing untagged vhs, were transfected into HeLa cells together with the GLuc expressing plasmid. As a negative control, a vhsH variant (vhs*) that has a point mutation in the open reading frame known to severely attenuate its activity [13] was also included. The resulting levels of secreted GLuc indicated that the vhs homologues divided into two groups – one that comprises vhsH, vhsB and vhsE, which were highly active for GLuc downregulation compared to vhs*, and one that comprises vhsV and vhsM, which had only modest GLuc downregulation compared to vhs* (Fig. 3a).

To explore this relative activity further, we next carried out a dose response of each of these vhs-expressing plasmids in comparison to the vhs*-expressing plasmid and measured secreted GLuc activity. This indicated that as little as 10 ng of transfected vhsB-expressing plasmid reduced GLuc activity by over fivefold, greater than that seen for vhsH or vhsE (Fig. 3b). A further dose response using even lower amounts of vhs plasmid (2 to 12 ng) indicated that as little as 2 ng of vhsB plasmid was able to reduce GLuc activity by over 70 % (Fig. 3c). The vhsB homologue was reproducibly more powerful at reducing GLuc expression than vhsH or vhsE homologues under the conditions employed in this assay. Given that vhsB is expressed at higher levels than the other homologues (Fig. 2a) this enhanced activity is likely to be due in part to higher levels of the vhsB protein in the cytoplasm of the cell.

The known effect of vhsH on cellular mRNA is as an endoribonuclease, and hence it is predicted that its activity on GLuc expression would be a consequence of enhanced decay of the GLuc mRNA. To determine the corresponding effect of the vhs homologues on the level of GLuc mRNA, transfections were carried out as above using a single dose of plasmid expressing untagged vhs, and the relative amount of GLuc mRNA was measured using RT-qPCR. This revealed that those vhs homologues that had greatly reduced GLuc expression at the protein level had also reduced the level of the GLuc transcript (Fig. 3d). Likewise, the co-expression of each of the vhs homologues with a plasmid expressing GFP had a similar effect on the GFP transcript (Fig. 3e), indicating that the differential activity of the homologues was unrelated to the reporter being tested.

### Differential effect of vhs homologues on PABPC1 relocalization to the nucleus

**Fig. 4.**
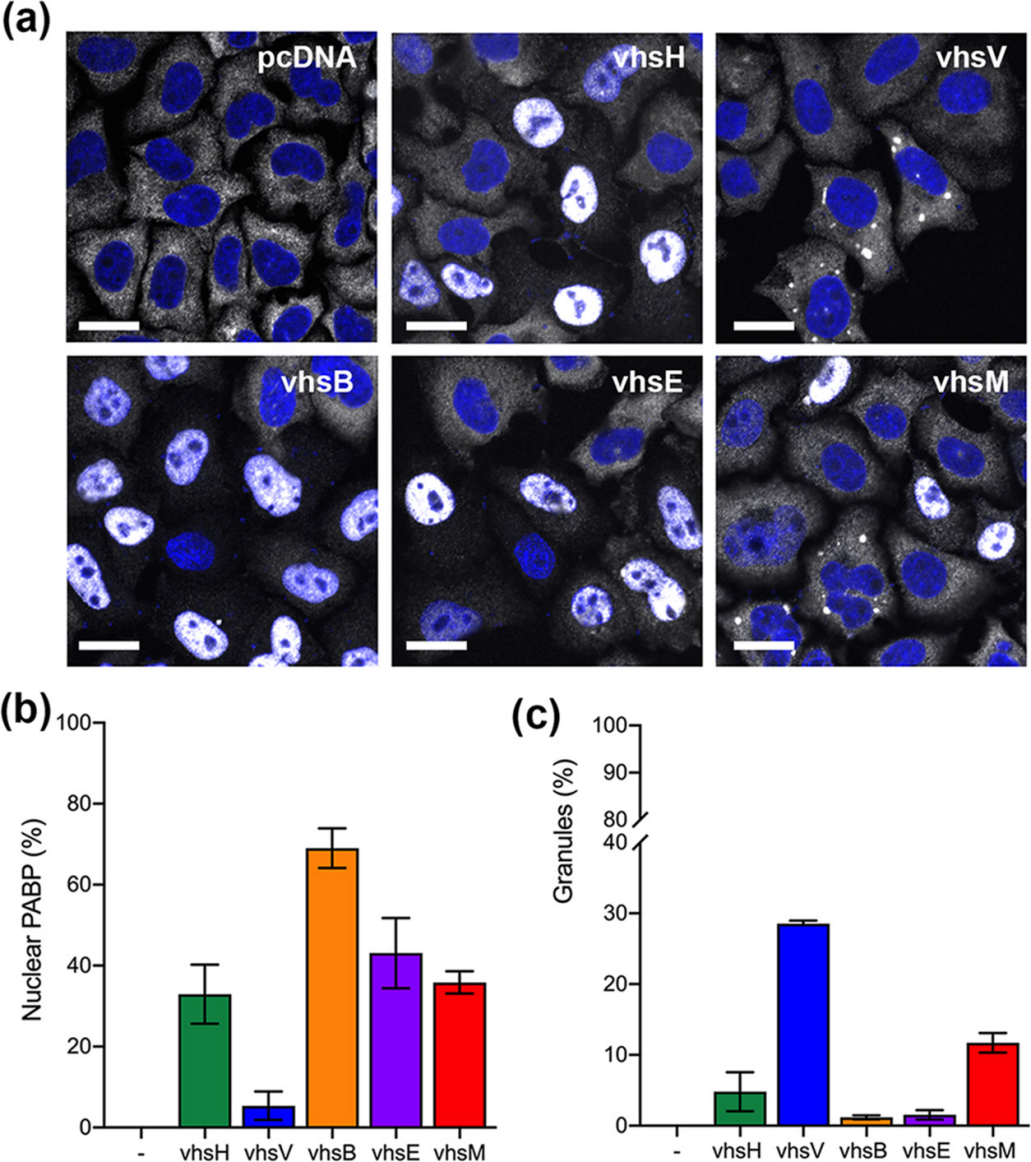
Differential effect of vhs homologue expression on PABPC1 localization. (**a**) HeLa cells grown on coverslips were transfected with plasmids expressing untagged vhs homologues, fixed 20 h later and stained for PABPC1 (white). Nuclei were stained with DAPI (blue) and cells were imaged on a NikonA1 confocal microscope. Scale bar=20 µm. (**b**) Cells with nuclear PABPC1 shown in (**a**) were counted in three independent experiments (> 150 cells in each). (**c**) Cells with cytoplasmic granules of PABPC1 in (**a**) were counted in two independent experiments (> 150 cells in each).

The expression of vhs is known to cause a change to the steady-state localization of the PABPC1 protein from cytoplasmic to nuclear, an activity that is proposed to reflect the enhanced turnover of mRNA in the cytoplasm and the concomitant release of PABPC1 to recycle to the nucleus [23,46]. To test the ability of the vhs homologues to alter the compartmentalization of PABPC1, we examined PABPC1 localization in cells expressing untagged vhs homologues, which indicated that PABPC1 was found in the nucleus of up to 60 % of the cell monolayer, with the percentage of cells containing nuclear PABPC1 broadly correlating with relative vhs activity: vhsB >vhsE> vhsH > vhsV (Fig. 4a, b). The one exception to this gradient was vhsM, which induced PABPC1 relocalization to the nucleus of over 30 % of cells despite causing only a modest reduction in GLuc activity (Fig. 4a, b, vhsM). Strikingly, the predominant effect of vhsV expression was not to relocalize PABPC1 to the nucleus but rather to induce PABPC1-containing cytoplasmic accumulations (Fig. 4a–c, vhsV), an outcome that was also present in combination with nuclear PABPC1 in vhsM expressing-cells (Fig. 4a, c, vhsM).

### Vhs homologues that do not induce mRNA downregulation induce the assembly of cytoplasmic G3BP1-containing RNA granules

**Fig. 5.**
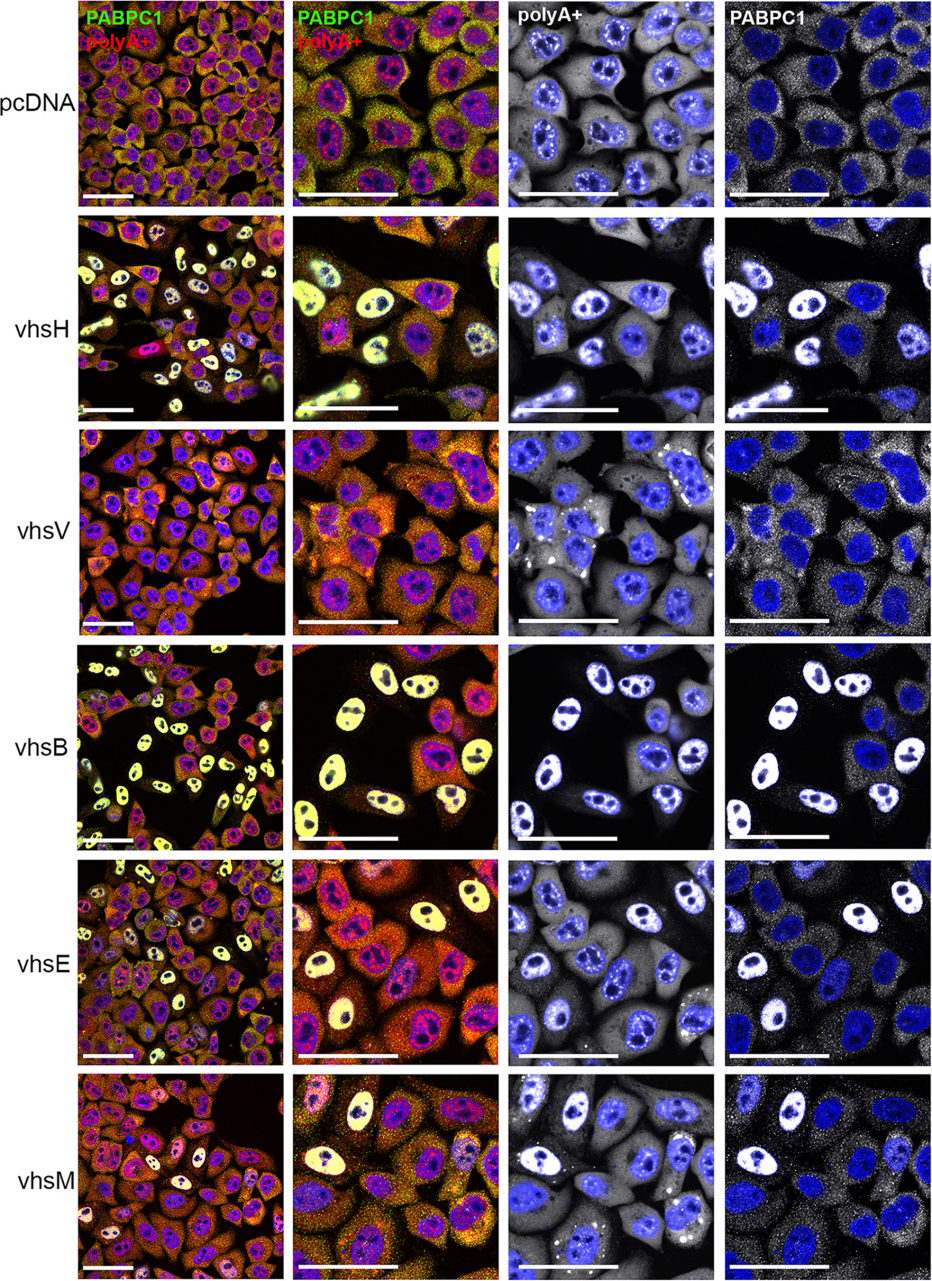
Differential effect of vhs homologues on the localization of polyadenylated RNA. HeLa cells grown on coverslips were transfected with plasmids expressing untagged vhs homologues, fixed 20 h later and processed first for poly(A)+ FISH (red) and second for PABPC1 immunofluorescence (green). Nuclei were stained with DAPI (blue) and cells were imaged on a NikonA1 confocal microscope. Scale bar=50 µm.

**Fig. 6.**
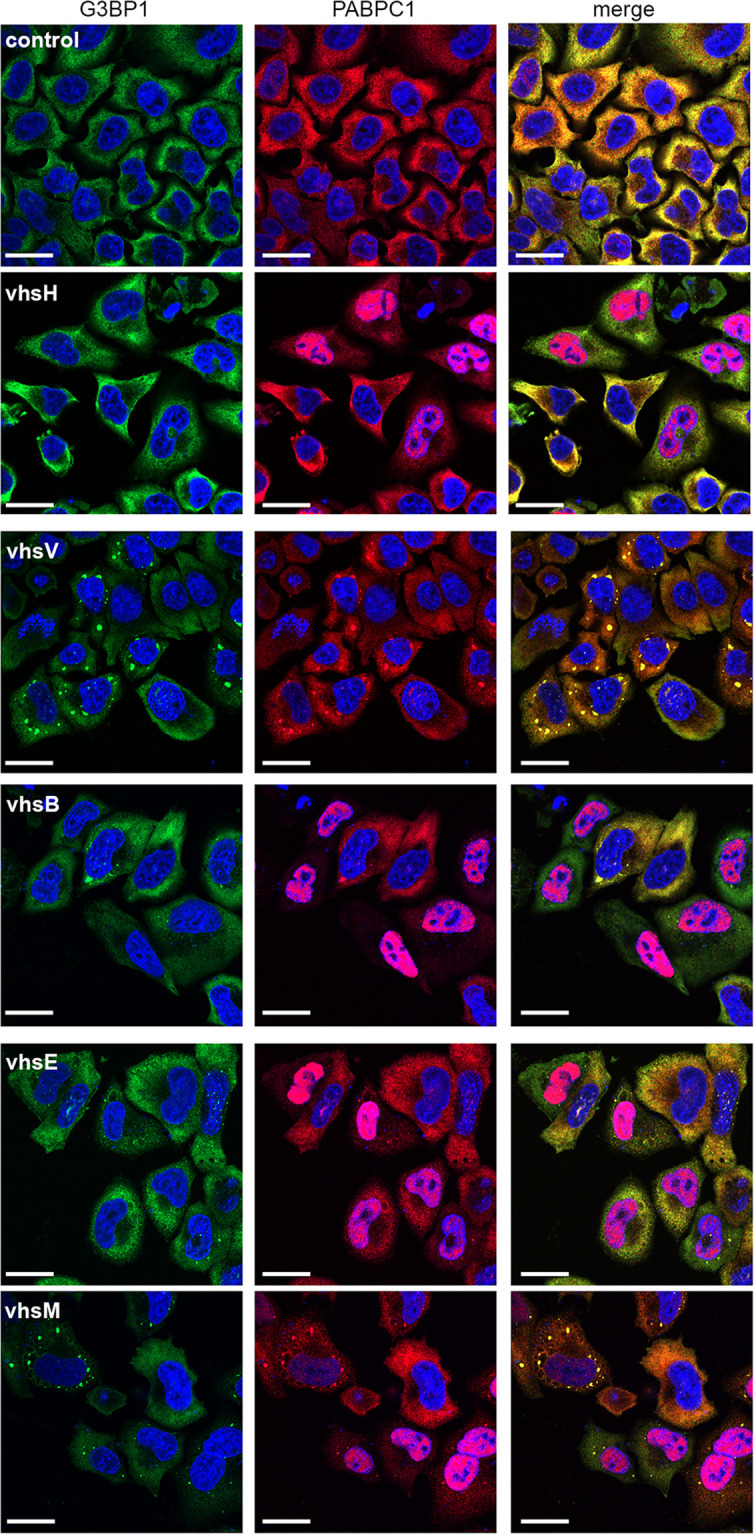
HeLa cells grown on coverslips were transfected with plasmids expressing vhs homologues, fixed 20 h later and stained for G3BP1 (green) and PABPC1 (red). Nuclei were stained with DAPI (blue) and cells were imaged on a NikonA1 confocal microscope. Scale bar=20 µm.

**Fig. 7.**
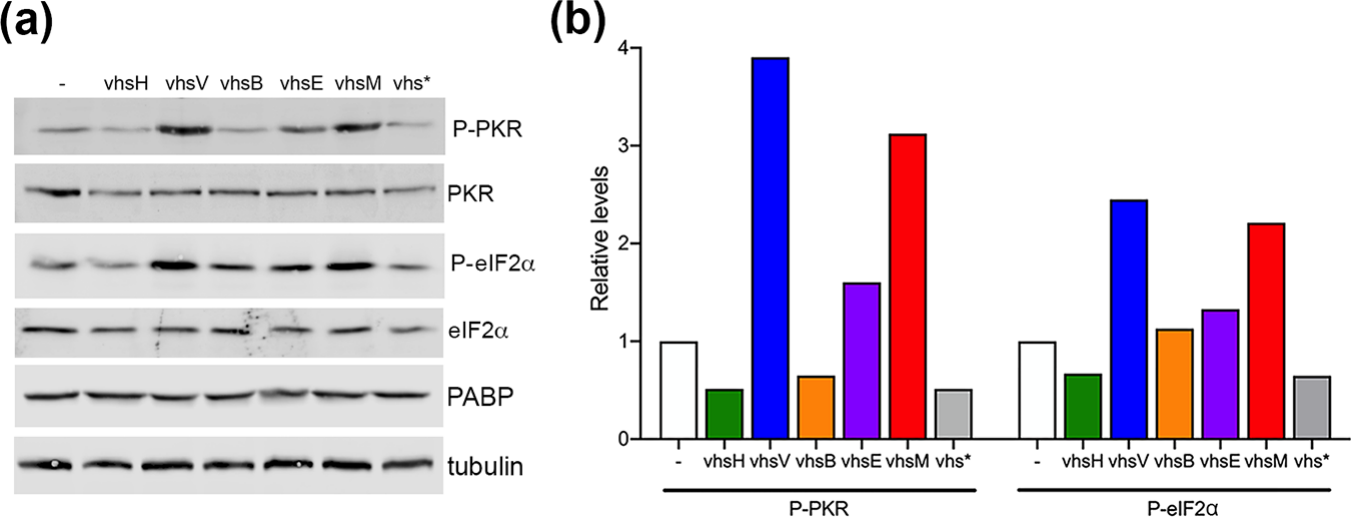
Induction of PKR and eIF2α phosphorylation correlates with SG formation by vhsV and vhsM. (**a**) Lysates of HeLa cells transfected with plasmids expressing each of the vhs homologues were analysed by SDS-PAGE and Western blotting with the indicated antibodies. (**b**) Quantification of the Western blots from one representative experiment [shown in (a)] using LI-COR Odyssey software. Phospho -PKR and -eIF2α were normalized to the total of each protein and subsequently to α-tubulin.

To determine the effect of vhs homologue expression on global mRNA, we utilized *in situ* hybridization of polyadenylated RNA using a fluorescently labelled oligodT probe. Cells incubated with oligodT were further stained for PABPC1 to correlate the global localization of polyA tails with the PABPC1 polyA tail-binding protein (Fig. 5). This revealed that PABPC1 localization correlated with that of poly(A)+ RNA, with vhsH, vhsB and vhsE inducing the cytoplasmic depletion and the nuclear accumulation of the poly(A)+ signal. However, in the case of vhsV and vhsM, poly(A)+ RNA accumulated in cytoplasmic granules. These RNA granules resemble stress granules (SGs), which are membraneless biocondensates formed when translation is stalled, and contain preinitiation translation complexes, 40 s ribosomal subunits and over 400 proteins including PAPBC1 and the classical SG marker, G3BP1 [47]. We therefore used G3BP1 alongside PABPC1 in cells expressing each of the vhs homologues to show that the RNA granules induced by expression of vhsV and vhsM also contained G3BP1, suggesting that they are formed as a consequence of stalled translation (Fig. 6). Furthermore, expression of vhsM and vhsV but not the other homologues also resulted in enhanced levels of both PKR and eIF2α phosphorylation suggesting that the RNA granules in these cells were a consequence of stress responses (Fig. 7a, b).

### All homologues of vhs induce global translational arrest

**Fig. 8.**
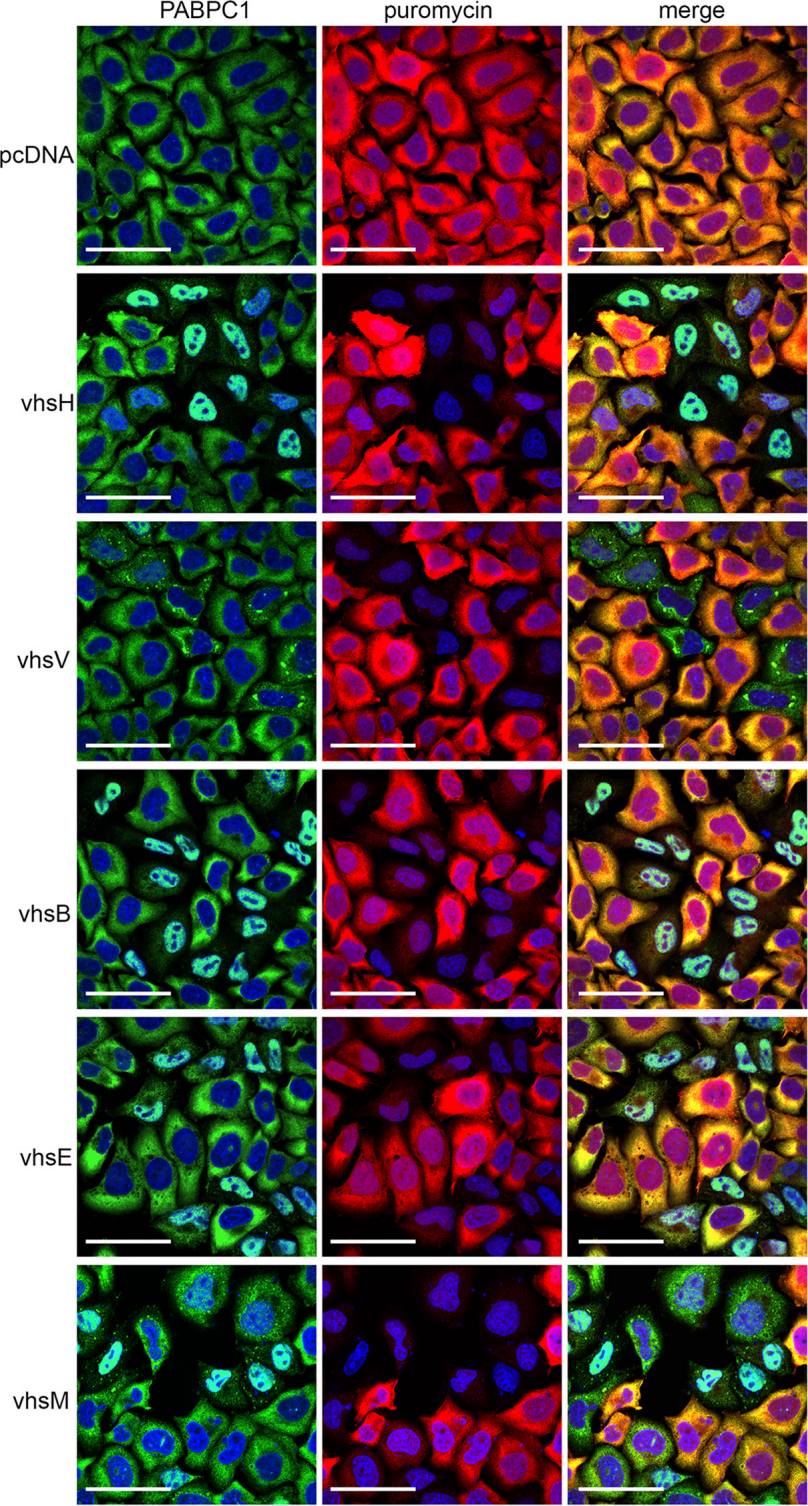
All vhs homologues induce translational blockade regardless of mRNA decay activity. HeLa cells grown on coverslips were transfected with plasmids expressing each of the vhs homologues. After 16 h, cells were incubated with puromycin for 10 mins before fixation and permeabilization. Cells were then stained with antibodies to PABPC1 (green) and puromycin (red), nuclei stained with DAPI (blue) and images acquired using a Nikon A1 confocal microscope. Scale bar=50 µm.

To measure translational arrest directly, we next carried out single-cell puromycin incorporation assays of HeLa cells expressing each of the untagged vhs homologues, and subsequently stained for both puromycin together with PABPC1, used here as a surrogate for vhs expression (Fig. 8). This indicated that in cells expressing vhsH, vhsB or vhsE, puromycin incorporation was greatly reduced in cells where PABPC1 was nuclear and hence where mRNA decay had occurred. However, in cells expressing vhsV or vhsM where cytoplasmic PABPC1 granules had formed, puromycin incorporation was also reduced to a similar degree. Hence, the two vhs homologues that are shown above to be defective at inducing mRNA degradation, instead appear to induce translational arrest through the formation of RNA granules.

## Discussion

In this study we have utilized a comparative biology approach to refine the functional pathway of the conserved alphaherpesvirus protein vhs, which in HSV1 has been shown to be an endoribonuclease. Using a combination of reporter assays and single-cell biological studies, we have shown that the vhs homologues fall into two groups: those that induce the degradation of mRNA resulting in translational shutoff, and those that only cause a block to translation. These unexpected results not only contribute to the ongoing molecular elucidation of vhs activity, but also help to unravel the potential role and mechanism of vhs in alphaherpesvirus infections other than HSV1.

It has generally been accepted that translational shutoff in HSV1 infection is a consequence of the global mRNA decay induced by vhsH. Indeed, the data presented here and elsewhere on ectopic expression of vhsH support that global RNA decay and translational shutoff go hand-in-hand in cells expressing not only vhsH, but also vhsB and vhsE. Nonetheless, vhsH does not cleave RNA indiscriminately but rather has been shown to be specific for mRNA, a mechanism that has been proposed to occur by binding to the translational initiation machinery at the 5′ end of the mRNA [18,21]. As such, this provides scope for vhsH protein to shut down translation prior to and without the need for mRNA decay. Intriguingly, the ectopic expression of the vhsV homologue did not induce decay of GLuc or GFP mRNA, nor did it induce the cytoplasmic depletion of polyadenylated RNA, yet it blocked global protein synthesis as measured by greatly reduced puromycin incorporation, as well as a significant reduction in GLuc activity. It would therefore be interesting to test if vhsV, retains the capacity to bind the translation initiation machinery while lacking endoribonuclease activity – at least when expressed in isolation of virus infection. If this is the case, then this may in some way explain the discrepancies in previous studies of vhsV ORF17 functionality [42,43]. Consequently, vhsB and vhsV appear to sit at two ends of the vhs endoribonuclease activity spectrum, exhibiting the greatest and least effect on polyadenylated RNA/PABPC1 relocalization, respectively. Intriguingly, vhsM exhibited a mixed phenotype, such that when expressed in HeLa cells, it had similar characteristics to vhsV, as it was unable to degrade GLuc or GFP mRNA but at least in some cells PABPC1 was relocalized to the nucleus. However, MDV is a virus that infects chickens, so it is likely that vhsM cannot function correctly in mammalian cells due to differences in important cellular factors. Hence, further studies in chicken cells will be needed to further define the activity of vhsM in an appropriate system. Taken together, these data lead us to propose that there could be two steps to vhs activity: (1) binding to the translational initiation machinery leading to translational blockade, and (2) the cleavage of associated mRNA leading to global mRNA decay; all homologues possess the ability to undertake step 1, but vhsV and to a large extent vhsM are defective in step 2. The fact that the metal ion binding residues in the vhsH active site are conserved across the homologues [18] suggests that these apparent differences in mRNA decay activity may be a consequence of the homologues engaging vital cellular factors with varying efficiency. Moreover, the full activity of at least some of these vhs homologues may require additional virus factors, exemplified by the regulation of vhsH by VP22 and VP16 [24,48].

It has been shown before that infection with HSV1 or HSV2 lacking vhs induces the assembly of SGs [49,50]. It has also been proposed that vhs is required to degrade dsRNA formed in infected cells, and in its absence, dsRNA is bound by PKR, which is activated to phosphorylate eIF2α and inhibit translation initiation. The downstream outcome of this would be the assembly of SGs in Δvhs-infected cells. Given this proposed role for vhs in blocking SG assembly, it is therefore counterintuitive that the activity of vhsV and vhsM homologues induce the assembly of RNA granules similar to canonical SGs and enhance the phosphorylation of PKR and eIF2α. One possible explanation for this outcome is that PKR is not activated directly by vhsV or vhsM expression, but that these vhs variants block translation directly by binding the initiation machinery, causing translational arrest and the downstream assembly of SGs. These SGs would then activate PKR and eIF2α phosphorylation, as shown by others [51,52].

One of the defining characteristics of vhsH is that, when introduced ectopically on a plasmid, it is expressed at very low levels [13]. Moreover, this low level of expression is maintained when vhsH is expressed as a GFP fusion protein [44], suggesting that it is an inherent feature of the vhsH ORF. We have previously described three reasons for this low level of vhs protein: first, because vhsH is an endoribonuclease, it could induce the degradation of its own transcript, thereby inducing a negative feedback loop to control its expression [13]. Second, we have also shown that unlike other transcripts, which generally accumulate in the cytoplasm, the vhsH transcript is inherently nuclear, providing an additional mechanism for regulation of vhs expression [13]. Third, we have also identified a short region of the vhsH ORF known as the inhibitory sequence (IS), which is fully transferable to other transcripts and reduces their expression [13]. Because fusion of GFP to vhsH abrogates vhs activity, it is likely that the reduced expression of vhsH-GFP is not due to self-induced degradation but is due either to the presence of the inhibitory sequence or the nuclear retention of its mRNA. It is therefore noteworthy that all homologues of vhs tested here also conferred a similarly low level of GFP expression when fused to the GFP ORF. Whether any or all of these homologues contain translation inhibitory sequences similar to that found in vhsH remains to be determined, but it is noteworthy that there was a gradient of expression of vhsB >vhsE>vhsM>vhsH> vhsV. The relatively high expression of vhsB in comparison to vhsH may partially explain its enhanced activity in the assays we have utilized, but this is yet to be confirmed.

The data presented here has allowed us to refine our overarching model for vhs activity. In this model, we propose that all vhs homologues tested would bind to mRNAs via the translation initiation machinery, resulting in translational stalling. In the absence of mRNA decay, translational stalling would lead to the assembly of cytoplasmic RNA granules, an amplification of the PKR response pathway, and a global shutdown of protein synthesis. However, endoribonuclease active vhs homologues would cleave associated mRNAs thereby inducing their degradation and release of PABPC1 to the nucleus, preventing SG assembly and PKR phosphorylation. This mRNA degradation would block the assembly of RNA granules induced by vhs, freeing up ribosomal subunits and relevant RBPs for further activity. While there are many steps of this pathway that still need to be defined, these data reveal the power of a comparative biology approach to understand not only how vhs functions but also key steps in the regulation of cellular mRNA decay and translational control.

## Abbreviations

BHV1: bovine herpes virus type 1
EHV1: equine herpesvirus type 1
eIF2α: eukaryotic initiation factor 2 alpha
FISH: fluorescent *in situ* hybridization
GFP: green fluorescent protein
GLuc: *gaussia* luciferase
HSV1: herpes simplex virus type 1
KSHV: Kaposi's sarcoma herpesvirus
MDV: Marek's disease virus
PABPC1: polyA binding protein C1
PKR: protein kinase R
SG: stress granule
vhs: virion host shutoff
VZV: varicella zoster virus
